# 23 Beyond the Bedside: Mediating Vacancy in a Specialty Educator Role

**DOI:** 10.1093/jbcr/irae036.023

**Published:** 2024-04-17

**Authors:** Stacey Richerbach, Tiffany Hockenberry, Kevin N Foster

**Affiliations:** Arizona Burn Center Valleywise Health, Phoenix, AZ; Arizona Burn Center Valleywise Health, Phoenix, AZ; Arizona Burn Center Valleywise Health, Phoenix, AZ

## Abstract

**Introduction:**

National recognition of burn nursing as a specialty is new, but nationwide challenges to recruitment and retention of qualified burn nurses are longstanding. As nursing shortages have reverberated throughout the US, the role of the nurse educator has become integral to onboarding success and continuing education. In a specialty such as burn, the role of the nurse educator is of immense importance. In 2021, the Burn Nurse Educator (BNE) at our facility transitioned from full to part-time. The vacancy in the BNE role proved difficult to fill. Rather than lower the requirements for the position, our team opted to bridge the gap with implementation of a staff-led Burn Education Committee.

**Methods:**

The part-time BNE and Burn Nurse Specialist worked in tandem as co-chairs and recruited committee members. Recruitment was initiated through word of mouth and formal advertisements in the weekly staff newsletter. Recruitment for the Burn Education Committee (BEC) was restricted to staff employed in the Burn Center, but remained open to various roles (e.g., registered nurses, certified burn techs, therapists, etc.). Upon recruiting members, the committee instituted bi-monthly meetings. A Learning Needs Assessment was disseminated to Burn Staff. Topics of interest for education were introduced within the committee, and members collaborated within the group to produce education in a variety of formats.

**Results:**

The BEC developed presentations, tipsheets and flyers, assisted with simulation and mock code events, and members aided in burn-specific course development. As the learning needs expressed by staff per the Learning Needs Assessment were satisfied, the BEC worked to develop education to aid with onboarding and continuing education. Two buckets of virtual lessons were developed for staff. In the first bucket, virtual modules included Introduction to Burn course and Burn Nurse Standards. The first bucket is completed by new staff during orientation and is scheduled to recur annually. The second bucket includes the virtual lessons: Intra-abdominal Pressure Monitoring, Management of Patients with Large Burns, Pediatric Fluid Resuscitation, Train of Four Peripheral Nerve Stimulation, and Warming Measures for Burn Patients. This recurring bucket is scheduled annually and completion is due before our busy season commences.

**Conclusions:**

Feedback from staff was positive, and interest in the committee increased as evidenced by additional staff joining the committee. Implementation of a staff-led BEC has revealed several benefits. Clinical staff have an expedited means of sharing education needs. Additionally, committee members have an expanded sense of purpose in the Burn Center and make an impact that spans beyond the bedside.

**Applicability of Research to Practice:**

This study demonstrates a novel and effective method to provide ongoing burn education to nursing staff in the face of limited resources.

